# The mediating and joint effects of depression in the association between cardiovascular health and frailty in middle-aged and elderly people: evidence from NHANES

**DOI:** 10.3389/fpsyt.2025.1578743

**Published:** 2025-04-15

**Authors:** Qiaoli Ma, Zhijuan Zou, Yanpeng Liu, Lei Zhang

**Affiliations:** ^1^ Department of Cardiology, Zibo Central Hospital, Zibo, Shandong, China; ^2^ Department of Orthopedic Trauma, Zibo Central Hospital, Zibo, Shandong, China; ^3^ Emergency Department, Zibo Central Hospital, Zibo, Shandong, China

**Keywords:** Life’s Essential 8, frailty, depression, NHANES, CVH

## Abstract

**Objective:**

This study investigates the correlation between Life’s Essential 8 (LE8), a cardiovascular health (CVH) metric, and frailty in middle-aged and elderly individuals in the United States, also examining how depression mediates this relationship.

**Methods:**

Participants from the NHANES 2005-2018 were analyzed for correlations between LE8 and frailty, along with the combined effects of LE8 and depression using multiple logistic regression. Dose-response relationships were assessed using restricted cubic splines (RCS), and mediation analysis explored depression’s role. Sensitivity and subgroup analyses were conducted for result stability.

**Results:**

The study included 8,982 participants, with 3,103 frailty events. A higher LE8 score was significantly associated with a reduced risk of frailty, with adjusted odds ratios for the medium and high CVH groups at 0.49 (95% CI: 0.40-0.58, p < 0.001) and 0.21 (95% CI: 0.13-0.33, p < 0.001), respectively. The RCS model showed a negative dose-response relationship. No significant association was found between LE8 and frailty in the depressed population, where depression mediated 32.13% of the relationship.

**Conclusion:**

Maintaining good cardiovascular health, as measured by LE8, is key to reducing frailty risk in middle-aged and elderly individuals. This underscores the importance of cardiovascular health assessments and targeted health programs to improve quality of life.

## 1 Introduction

Over the past decade, global aging has experienced exponential growth due to factors such as population growth, a declining birth rate, and increasing life expectancy. The World Health Organization (WHO) estimates that by 2030, one in six people worldwide will be aged 60 or older, with a significant increase in the proportion of the population over 60 (1). By 2050, it is projected that 21% of the global population will be aged 60 or older, with this trend being particularly evident in China (2).In China, the population aged 60 or older is expected to increase from 130 million in 2000 to 370 million by 2050, rising from 11% to 26% of the total population (3).This trend has given rise to a new challenge: frailty among the elderly.

Frailty is characterized by reduced physiological reserves, increased vulnerability, and impaired stress tolerance. It is a nonspecific condition influenced by various factors (4). Among the elderly, frailty is a geriatric syndrome that represents an intermediate stage between independent living and disability or death. The acceleration of global population aging has led to a rapid increase in frailty incidence (5).A global meta-analysis involving 755,497 participants from 62 countries and regions found that the frailty prevalence among the elderly was 12% (6).The prevalence of frailty among the Chinese population is approximately 3.1% (7). A higher prevalence, ranging from 15% to 25%, has been observed among middle-aged and elderly individuals (8, 9). Therefore, reducing the incidence of physical frailty is of significant importance in public health. Frailty often significantly increases the incidence of cardiovascular events in middle - aged and elderly populations, and this trend is more prevalent among the Chinese population. A Chinese Longitudinal Healthy Longevity Cohort Study shows that frail elderly people exhibit a higher risk of CVD and all - cause mortality (10).

Cardiovascular health (CVH) is strongly related to the health status of the elderly. Cardiovascular disease (CVD) remains one of the leading causes of death among the elderly worldwide. In 2019, global data indicated that approximately 17.9 million people died from cardiovascular diseases, representing 32% of total deaths. Of these deaths, 85% were due to myocardial infarction and cerebrovascular accidents. The majority of CVD deaths occurred in low- and middle-income countries (11).This includes coronary heart disease, cerebrovascular disease, peripheral artery disease, rheumatic heart disease, and congenital heart disease (12). Elderly individuals often have multiple CVD risk factors, including smoking, obesity, hypertension, and high cholesterol (13, 14). Additionally, the elderly are more susceptible to malignant tumors and respiratory diseases (15), increasing all-cause mortality in this population. There is a significant association between depression and the severity of frailty in middle-aged and elderly individuals. Studies have shown that frailty is more severe in patients with depression than in non-depressed individuals (16). Depression not only affects mood and psychological well-being, but also exacerbates frailty by reducing physical activity and influencing behaviors such as diet and sleep (17).

In 2010, the American Heart Association (AHA) introduced “Life’s Simple 7” (LS7), a framework designed to promote CVH by assessing cardiovascular risk behaviors and indicators (18). CVH significantly impacts human health. Previous studies have shown that individuals with higher stress and depression levels tend to have lower LS7 scores (19), Ideal CVH indicators are significantly associated with ischemic heart disease (IHD) (20), and higher LS7 scores are linked to a reduced risk of CVD and all-cause mortality (21). In 2022, the American Heart Association (AHA) introduced the Life’s Essential 8 (LE8) score, an enhanced algorithm for assessing CVH (22, 23).Compared to the LS7 score, CVH consists of two components: healthy behaviors (e.g., nutrition, physical activity, nicotine exposure, and sleep health) and health factors (e.g., body mass index, non-high-density lipoprotein cholesterol, blood sugar, and blood pressure) (24). Previous studies using the LS7 have shown that ideal CVH can reduce cardiovascular diseases and mortality. However, its relationship with frailty remains under - explored. In addition, the expanded scope of LE8 provides a more comprehensive assessment of CVH (25). Some studies suggest that LE8 is significantly associated with the risks of chronic kidney disease, depression, and CVD-related mortality. This enhances its predictive ability for other diseases such as depression. However, the mediating role of depression between CVH and frailty has not been fully discussed.

Given the global aging of the population and the high prevalence of mental illnesses, it is necessary to investigate the mediating and joint effects of depressive symptoms between CVH and frailty among middle - aged and elderly individuals. However, it remains unclear whether “Life’s Essential 8” is associated with the risk of frailty in middle-aged and elderly individuals, or whether depression mediates the relationship between LE8 and frailty. This study aims to preliminarily explore this association through a large cross-sectional study conducted in the United States.

## 2 Materials and methods

### 2.1 Data sources and study population

**Figure 1:**
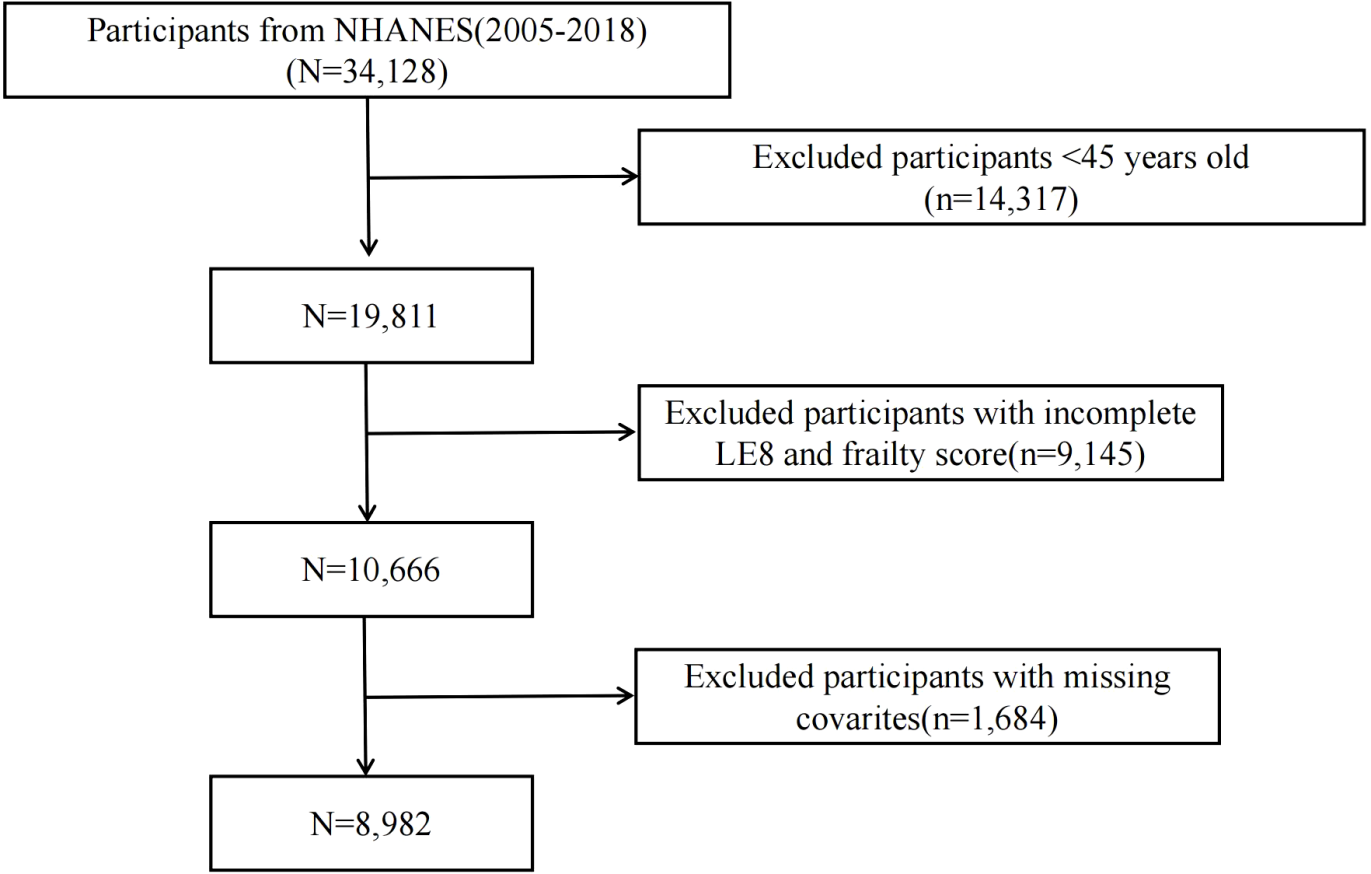
The flowchart in selecting the studying participants.

The National Health and Nutrition Examination Survey (NHANES) is a regular survey that uses interviews and physical examinations to assess the health and nutritional status of non-hospitalized individuals in the United States. All population data are publicly available at https://wwwn.cdc.gov/nchs/nhanes/Default.aspx. The NHANES protocol was approved by the National Health Statistics Research Ethics Review Board, and informed consent was obtained from all participants. This study was exempt from ethical review by the hospital’s ethics committee. This retrospective cohort study included 8,982 individuals aged ≥ 45 years from the NHANES database, spanning 7 cycles (2005–2018). Individuals with missing data were excluded. The participant registration flowchart is shown in **Figure 1**.

### 2.2 Measurement of life’s essential 8

The American Heart Association (AHA) recently introduced Life’s Essential 8 (LE8) as a method for assessing cardiovascular health (CVH). CVH consists of two components: health behaviors (diet, physical activity, nicotine exposure, and sleep health) and health factors (BMI, non-HDL cholesterol, blood glucose, and blood pressure). The total LE8 score was calculated by averaging the ratings for each of the 8 indicators. Standard protocols were used to assess the aforementioned measurements, and the detailed procedure for calculating each participant’s CVH metric is outlined in **Supplementary Table 1**, based on the AHA Presidential Advisory (26). The CVH metric ranges from 0 to 100. The LE8 and its scale scores were calculated by averaging the 8 metric scores. Thus, the LE8 score range also spans from 0 to 100. The LE8 score was categorized into three groups according to the AHA’s recommendations: low (LE8 < 50), moderate (50 ≤ LE8 < 80), and high (LE8 ≥ 80) (27).

### 2.3 Assessment of depression symptoms

Depressive symptoms were assessed using the 9-item Patient Health Questionnaire (PHQ-9) and antidepressant use. The PHQ-9 was used as a brief, self-reported measure of depressive symptoms over the past two weeks (28). It is a validated screening tool for major depressive disorder (MDD) and other depressive symptoms. When a cut - off score of ≥10 is used, its sensitivity is 88% and its specificity is 88% (29). The 9 items include anhedonia, depressed mood, sleep disturbances, fatigue, appetite changes, low self-esteem, attention problems, psychomotor disturbances, and suicidal ideation. Each item on the scale is scored from 0 (not at all) to 3 (nearly every day). The total PHQ-9 score ranges from 0 to 27, with a score of ≥ 10 indicating significant depressive symptoms (30).

### 2.4 Assessment of frailty

Frailty results from progressive cellular damage, leading to a decline in organ system function and a reduced ability to restore balance after stressful events (31). We used the Frailty Index (FI) to assess the level of frailty. This index consists of 49 variables covering multiple systems, including features such as cognition, dependency, depressive symptoms, comorbidities, general health status, hospital utilization, physical performance, body measurements, and laboratory test values (32–34). To be eligible, participants must complete at least 80% (approximately 40 items) of the 49 frailty items in the survey. Values were assigned on a scale from 0 to 1 based on the severity of defects (see **Supplementary Table 2**). The FI is calculated by dividing the sum of defect scores obtained by participants by the total possible defect score.When the Frailty Index (FI) reaches or exceeds 0.21, it is considered a key threshold for frailty. Conversely, if the FI is less than 0.21, it is classified as non-frail (35).

### 2.5 Covariates

To minimize the influence of confounding factors, we included demographic and health-related variables in the analysis. The demographic covariates included age (continuous), gender (male, female), race (Non-Hispanic White, Mexican American, Other Hispanic, Non-Hispanic Black, and Other Race, including multiracial), marital status (married, widowed, divorced, separated, unmarried, or living with a partner), educational attainment (below high school, high school, and post-high school education), and poverty-to-income ratio (PIR: ≤1.30, 1.31-3.49, and ≥3.50; a lower PIR indicates a higher risk of poverty) (36). Health-related variables included hypertension (no/yes), diabetes (no/yes), cancer (no/yes), CVD risk (no/yes), and alcohol consumption status (never, past, or current). The proportion of missing data for the total population is shown in **Supplementary Figure 1**.

### 2.6 Statistical analysis

This study used weighted methods for statistical analysis. Categorical variables are presented as counts and percentages (%), while continuous data are expressed as means with standard deviations (SD). Weighted chi-square tests were used for categorical variables, and weighted Mann-Whitney U tests were applied to compare continuous variables across different LE8 groups. Weighted binary logistic regression was used to investigate the association between LE8 and frailty, with results expressed as odds ratios (ORs) and 95% confidence intervals (CIs). Three models were constructed: Model 1 (unadjusted), Model 2 (adjusted for demographic characteristics: age, gender, race, marital status, educational attainment, and poverty-to-income ratio), and Model 3 (adjusted for health-related factors: hypertension, diabetes, cancer, CVD, and alcohol consumption). Three-knot restricted cubic spline (RCS) regression models were used to explore the non-linear relationship between LE8 and frailty, and to assess the linear dose-response relationship (37). We also examined the association between different components of LE8 and frailty.

A sensitivity analysis was conducted to assess the stability of our results. The R “mice” package was used to perform 10 multiple imputations for participants with missing covariates, with results summarized using Rubin’s rules (38). Inverse probability weighting was used to balance the distribution differences of covariates across different LE8 groups (39). Middle-aged and elderly participants with a history of cancer and cardiovascular diseases were excluded to eliminate the influence of disease history on frailty. Additionally, the cut-off value for the frailty index was set at 0.25 for sensitivity analysis (40). Subgroup analyses were conducted to ensure the consistency of results across different subgroups, including age, gender, race, education, marital status, PIR, and alcohol consumption. We examined the mediating role of depression in the relationship between LE8 and frailty, and its combined effect with LE8. Notably, in the mediation analysis, LE8 was the exposure variable, depression score was the continuous mediating variable, and frailty was the outcome variable, with a mediation model constructed for statistical analysis. This was performed using the R package “mediation” and the quasi-Bayesian method (41, 42). All statistical analyses were performed using R software (version 4.4.1), and a P < 0.05 was considered statistically significant. All statistical tests were two-tailed.

## 3 Results

### 3.1 The baseline characteristics of participants

Among the 8,982 middle-aged and elderly participants, the average age was 66.81 ± 9.35 years (**Table 1**). In the study cohort, 51.0% were female, 54.4% were non-Hispanic White, and 48.9% were college graduates. 55.8% of the study population were married. 29.6% had a poverty-to-income ratio (PIR) ≥ 3.5. 38.8% currently reported mild alcohol consumption. 58.9% had hypertension, 24.4% had diabetes, and 19.2% had cancer. Based on the LE8 score, 19.51% of the participants had low CVH, 70.64% had moderate CVH, and 9.85% had high CVH. Statistically significant differences were found in age, gender, ethnicity, education, marital status, PIR, hypertension, diabetes, and cancer across different LE8 groups.

**Table 1:** Baseline characteristics of participants by different life’s essential 8 levels.

Variables	Overall (n=8982)	Low (n=1752)	Moderate(n=6345)	High(n=885)	*P*
Age,year, (mean (SD)	66.81 (9.35)	64.91 (9.56)	67.21 (9.30)	67.70 (8.84)	<0.001
Gender					
Male	4397 (49.0)	790 (45.1)	3200 (50.4)	407 (46.0)	<0.001
Female	4585 (51.0)	962 (54.9)	3145 (49.6)	478 (54.0)	
Ethnic[N(%)]					
Non-Hispanic White	4883 (54.4)	831 (47.4)	3462 (54.6)	590 (66.7)	<0.001
Others^a^	4099 (45.6)	921 (52.6)	2883 (45.4)	295 (33.3)	
Education[N(%)]					
<High school	2346 (26.1)	648 (37.0)	1592 (25.1)	106 (12.0)	<0.001
High school	2245 (25.0)	470 (26.8)	1632 (25.7)	143 (16.2)	
>High school	4391 (48.9)	634 (36.2)	3121 (49.2)	636 (71.9)	
Marital status[N(%)]					
Married	5013 (55.8)	829 (47.3)	3592 (56.6)	592 (66.9)	<0.001
Others^b^	3969 (44.2)	923 (52.7)	2753 (43.4)	293 (33.1)	
PIR[N(%)]					
≤1.3	2665 (29.7)	752 (42.9)	1794 (28.3)	119 (13.4)	<0.001
1.3-3.5	3658 (40.7)	722 (41.2)	2638 (41.6)	298 (33.7)	
≥3.5	2659 (29.6)	278 (15.9)	1913 (30.1)	468 (52.9)	
Drinking[N(%)]					
Never	1345 (15.0)	242 (13.8)	962 (15.2)	141 (15.9)	<0.001
Former	2257 (25.1)	593 (33.8)	1537 (24.2)	127 (14.4)	
Mild	3484 (38.8)	499 (28.5)	2515 (39.6)	470 (53.1)	
Moderate	1026 (11.4)	178 (10.2)	738 (11.6)	110 (12.4)	
Heavy	870 (9.7)	240 (13.7)	593 (9.3)	37 (4.2)	
Hypertension[N(%)]					
No	3690 (41.1)	456 (26.0)	2632 (41.5)	602 (68.0)	<0.001
Yes	5292 (58.9)	1296 (74.0)	3713 (58.5)	283 (32.0)	
Diabetes[N(%)]					
No	6789 (75.6)	915 (52.2)	5010 (79.0)	864 (97.6)	<0.001
Yes	2193 (24.4)	837 (47.8)	1335 (21.0)	21 (2.4)	
Cancer[N(%)]					
No	7260 (80.8)	1491 (85.1)	5081 (80.1)	688 (77.7)	<0.001
Yes	1722 (19.2)	261 (14.9)	1264 (19.9)	197 (22.3)	

^a^including Mexican American, Other Hispanic, Non-Hispanic Black and Other Race - Including Multi-Racial. ^b^including Widowed, Divorced, Separated, Never married and Living with partner.

### 3.2 Association of the LE8 with frailty

**Figure 2:**
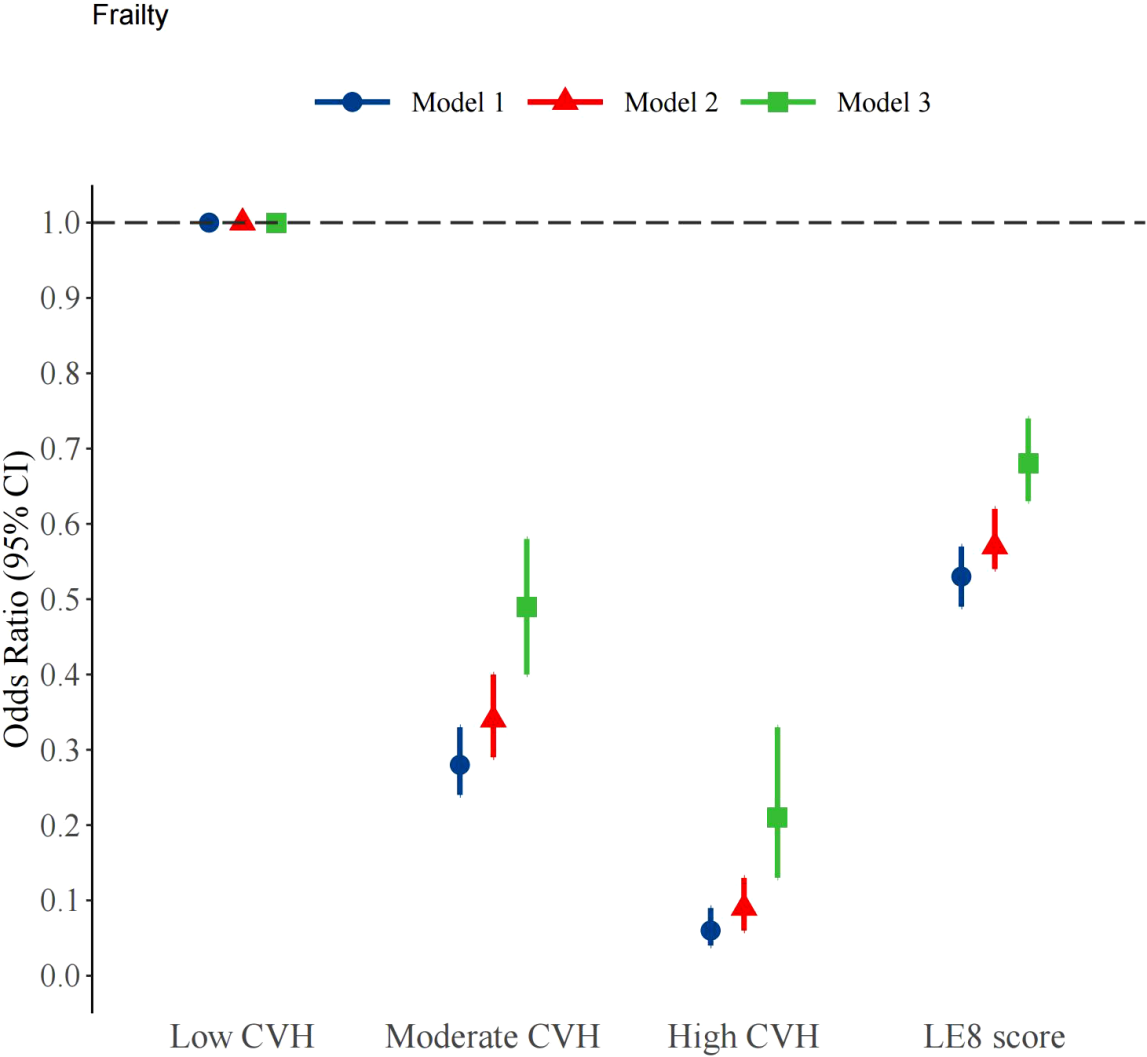
Survey weighted association of Life’s Essential 8 scores with frailty. Notes: Model 1 was unadjusted; Model 2 was adjusted for age, gender, ethnicity, education,marital status and PIR; Model 3 was additionally adjusted for CVD, hypertension, cancer, drinking and diabetes. LE8 score: Life’s Essential 8 scores, as a continuous variable, calculated per 10 points increase.

**Figure 3:**
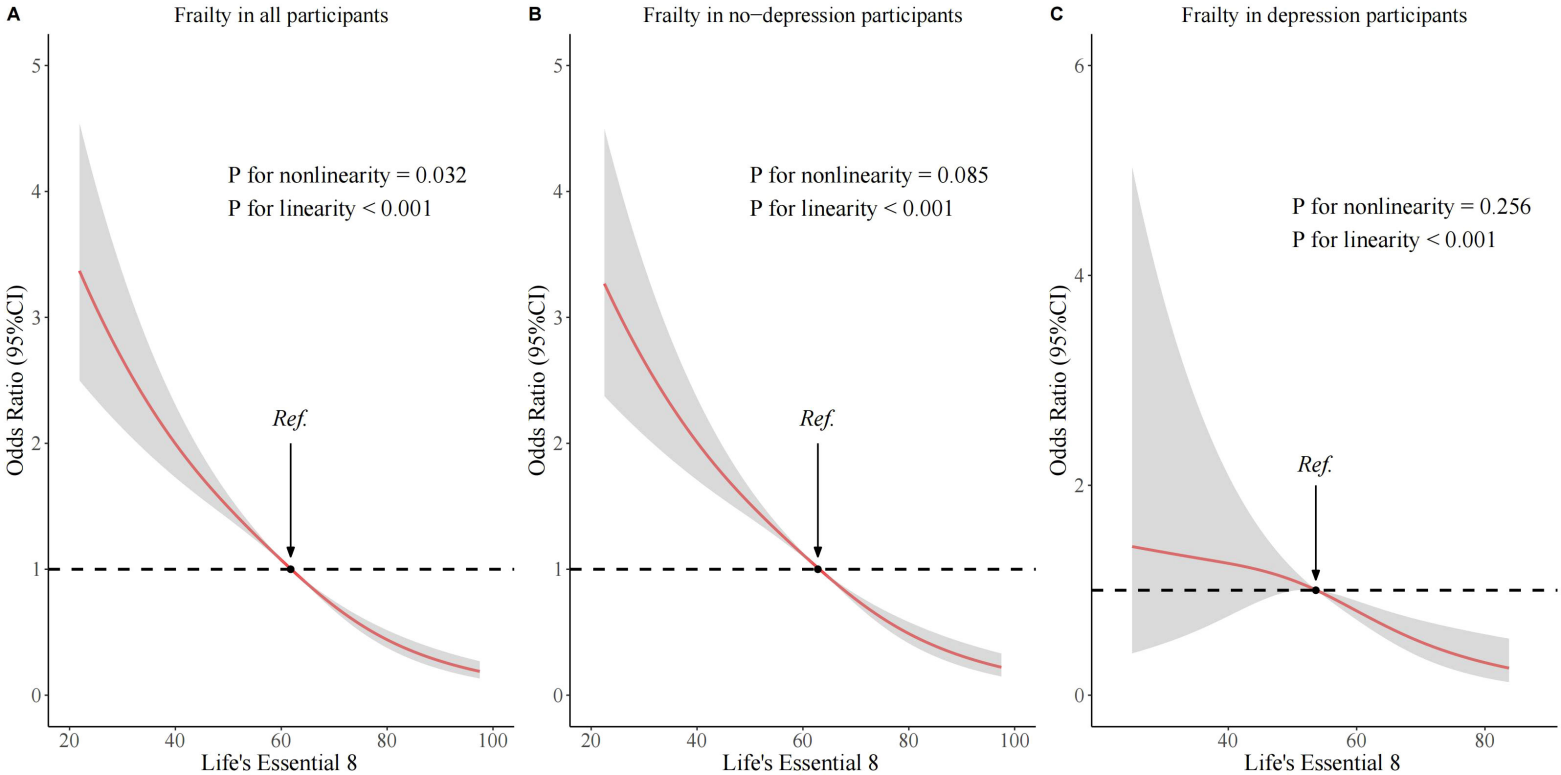
Associations of Life’s Essential 8 scores with frailty according to RSC regression. **(A)** in the all participants; **(B)** in the no-depression participants; **(C)** in the depression participants. The odds ratio and 95% CI were calculated by adjusting for all covariates.

As shown in **Supplementary Table 3**, three different models, accounting for a wide range of potential confounding factors, were used to explore the relationship between LE8 and frailty. In the model adjusted for all covariates, an association between LE8 and frailty was observed (**Figure 2**). Using low CVH as the reference group, the ORs for moderate and high CVH were 0.49 (95% CI: 0.40 - 0.58, p < 0.001) and 0.21 (95% CI: 0.13 - 0.33, p < 0.001), respectively. The trend test was also statistically significant (P for trend < 0.001). When LE8 was treated as a continuous variable, the results showed that in Model 3, each 10-point increase in the LE8 score was associated with a 32% reduction in the odds of frailty. Maintaining a high CVH score is significantly associated with frailty among middle - aged and elderly individuals. The dose-response relationship between the LE8 score and frailty was assessed using a three-knot restricted cubic spline (RCS) regression model, adjusting for all covariates (**Figure 3A**). The results showed a significant non-linear association between the LE8 score and the risk of frailty (p for non-linearity = 0.032). As the LE8 score increased, the risk of frailty decreased.

### 3.3 The mediating and joint effects of depression

**Figure 4:**
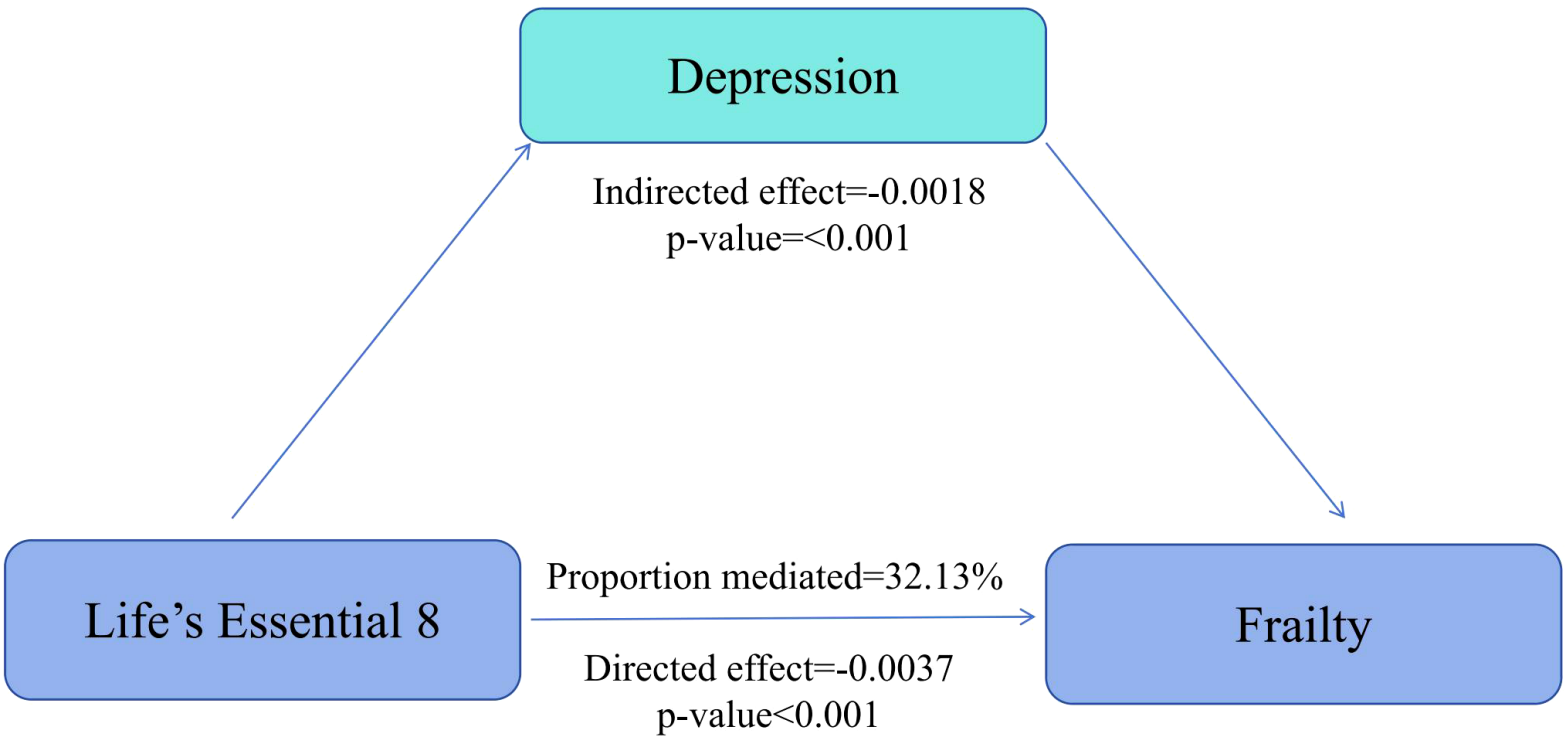
Mediation effect of depression for the association of LE8s with frailty. Adjusted for all covariates.

As shown in **Supplementary Table 4**, three different models were used to explore the combined effect of depression and LE8. Among participants without depression, an association between LE8 and frailty was observed. Using low CVH as the reference group, the ORs for moderate and high CVH were 0.49 (95% CI: 0.40 - 0.60, p < 0.001) and 0.21 (95% CI: 0.13 - 0.34, p < 0.001), respectively. However, among participants with depression, no significant association was found between different LE8 classifications and frailty. Using low CVH as the reference group, the ORs for moderate and high CVH were 0.59 (95% CI: 0.27 - 1.15, p = 0.162) and 0.55 (95% CI: 0.10 - 2.99, p = 0.486), respectively. The combined relationship between depression and LE8 with frailty showed that in Model 3, compared with the low CVH/depression group, moderate CVH/depression and high CVH/depression were not statistically associated with frailty, while low, moderate, and high CVH/non-depression were significantly associated with a reduced risk of frailty. The mediation analysis indicated that the depression score mediated 32.13% of the association between LE8 and frailty (**Figure 4**). Depressive symptoms serve as mediating factors between CVH and frailty in middle - aged and elderly people. The dose-response relationship between the LE8 score and frailty indicated that among participants with depression and those without depression, there was no significant non-linear association between the LE8 score and frailty, with non-linear p-values of 0.085 and 0.256, respectively (**Figures 3B, C**).

### 3.4 Subgroup and interaction analysis between LE8 scores and frailty

**Figure 5:**
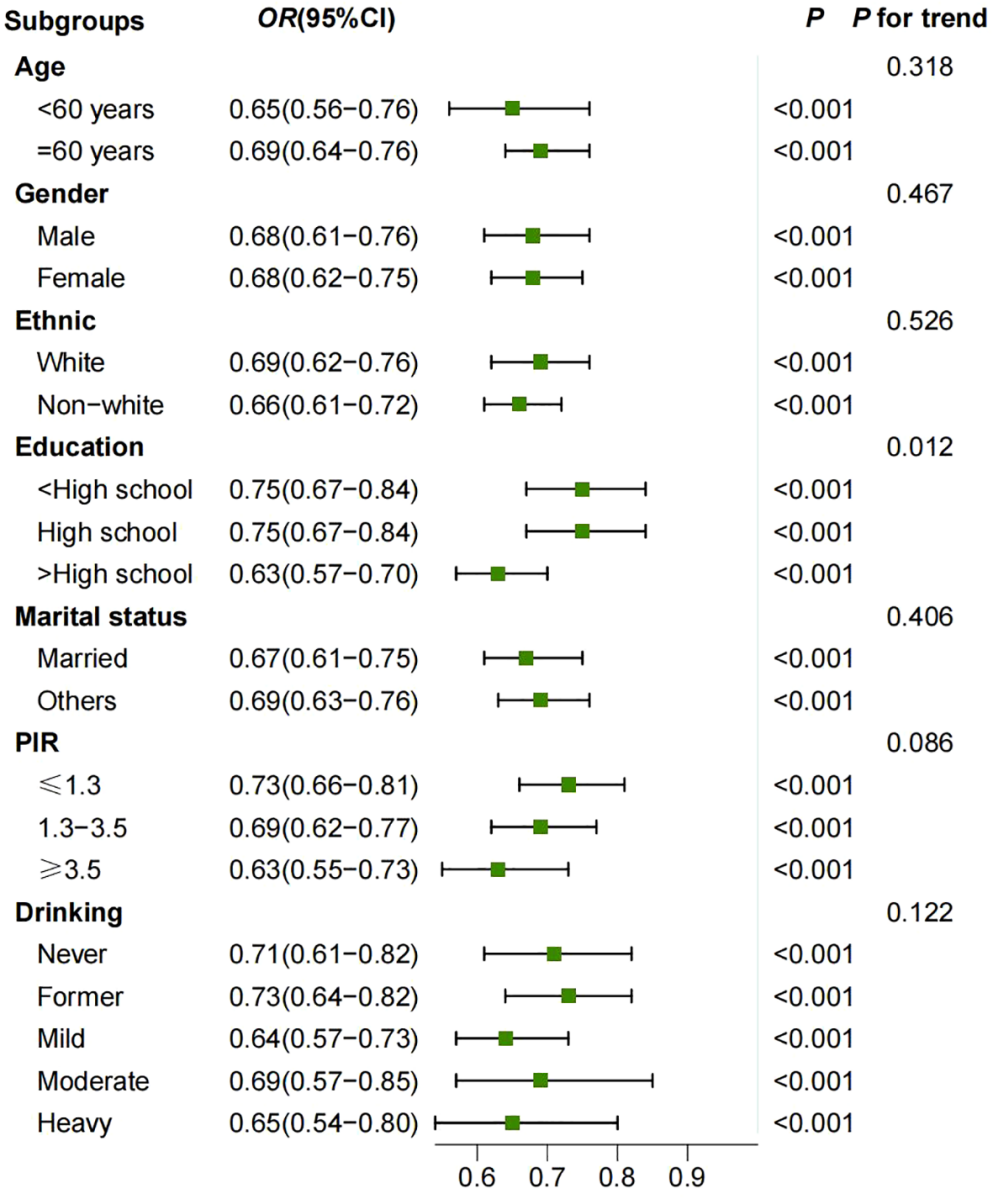
Survey weighted association of Life’s Essential 8 scores with frailty in subgroups. Model was adjusted for all covariates.

To further investigate the stability of the association between LE8 scores and frailty, subgroup analyses were conducted based on age (45-60 years and ≥ 60 years), gender (male and female), education level (< high school, high school, and > high school), ethnicity (non-Hispanic white and others), marital status (married and others), PIR (≤ 1.3, 1.3 - 3.5, and ≥ 3.5), and alcohol consumption (never, former, mild, moderate, and heavy). The results are shown in **Figure 5**. The results of the subgroup analyses were adjusted for all covariates. The results showed a significant association between LE8 and frailty in all subgroups. An interaction was found between education subgroups and LE8, with a p-value of 0.012; no significant interactions were observed in other subgroups.

### 3.5 Sensitivity analysis

**Figure 6:**
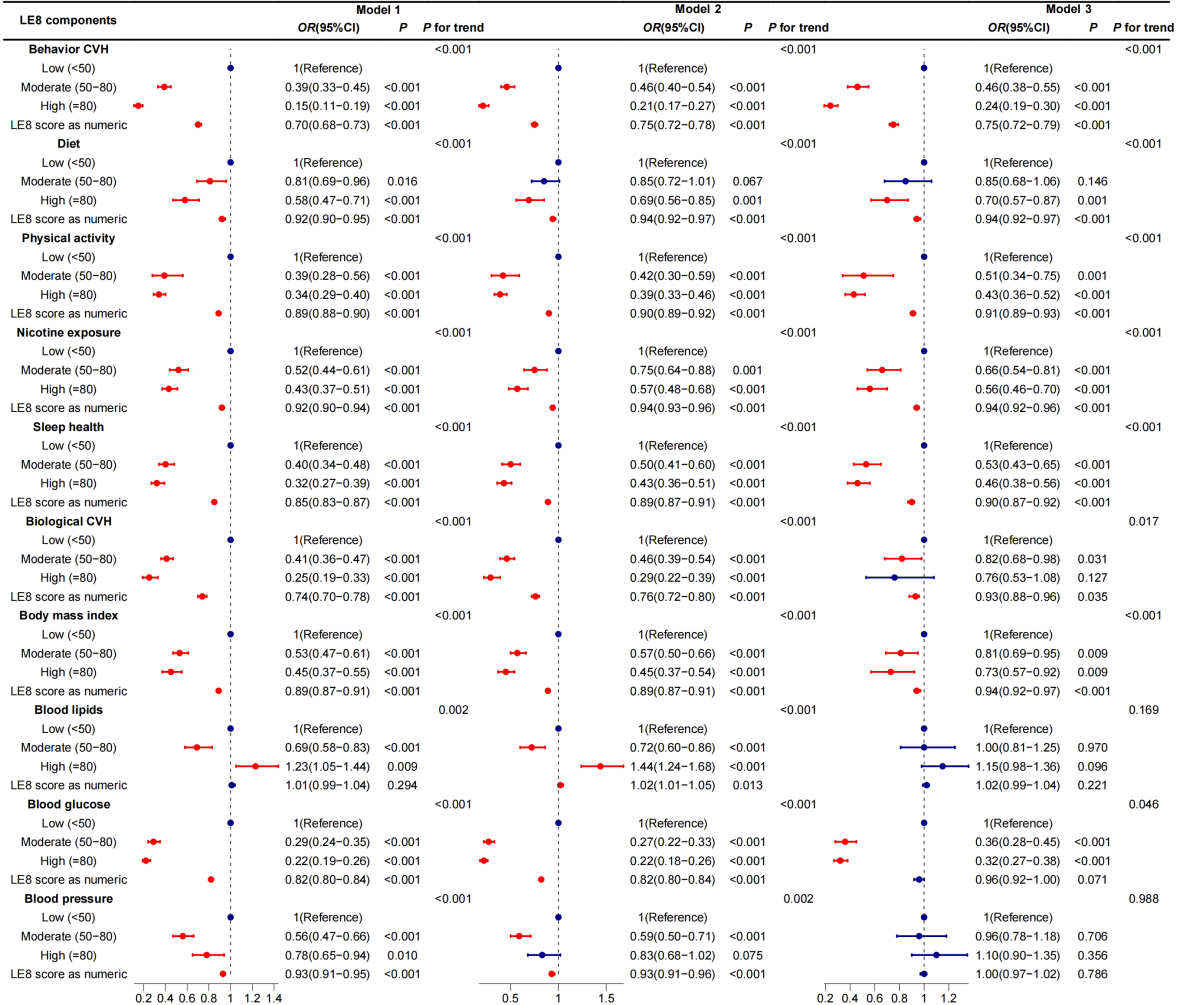
Survey weighted association of LE8’s components with frailty in subgroups. Model 1 was unadjusted; Model 2 was adjusted for age, gender, ethnicity, education,marital status and PIR; Model 3 was additionally adjusted for CVD, hypertension, cancer, drinking and diabetes. LE8 score: Life’s Essential 8 scores, as a continuous variable, calculated per 10 points increase.

Multiple sensitivity analyses were conducted to confirm the robustness of the association between LE8 and frailty. These included performing 10 multiple imputations, using IPTW to balance distribution differences of covariates across LE8 groups, setting 0.25 as the cut-off value for the frailty index, and excluding participants with a history of cancer and CVD at baseline. The results are shown in **Supplementary Tables 5–9**. The results were consistent with previous analyses, showing a significant association between LE8 and frailty. Additionally, separate analyses of different LE8 components were conducted to explore their association with frailty. The results showed that, after adjusting for three different models, the four behavioral health factors had a stronger association with frailty. The detailed results are shown in **Figure 6**.

## 4 Discussion

In this nationally representative cross-sectional study of middle-aged and elderly individuals in the United States, we found a significant association between a higher LE8 score and frailty. This association exhibited a dose-response relationship: as the LE8 score increased, the risk of frailty significantly decreased. Subgroup and multiple sensitivity analyses were conducted to verify the stability of the results. Additionally, we examined the combined and mediating effects of depression on the relationship between LE8 and frailty. The results showed that, among middle-aged and elderly participants with depression, a higher LE8 score was not significantly associated with frailty, and the depression score mediated 32.13% of the relationship between LE8 and frailty. These results highlight the importance of LE8 in managing frailty among the elderly.

Previous studies have highlighted the important role of LE8 in elderly health. Yang et al. found that a low CVH score was associated with a higher risk of eye diseases (43).Gou et al. demonstrated that an increase in the LE8 score in middle-aged and elderly individuals was a protective factor for MetS, with this association potentially mediated by biological aging. This suggests that LE8 may reduce the risk of MetS by improving aging (44).Cai et al. found that LE8 was negatively and non-linearly associated with the risk of AAC in the middle-aged and elderly population (45). Furthermore, the LE8 score is significantly associated with cardiovascular health and mortality among the elderly (46). Our study is consistent with previous research in terms of direction. Better CVH reduces the risk of frailty. Maintaining good CVH helps improve the quality of life and extend the healthy lifespan of the elderly.

A balanced diet supplies the body with a variety of essential nutrients. For example, adequate protein supports muscle protein synthesis and prevents muscle atrophy (47); Additionally, abundant antioxidants protect cells from free radical damage and help maintain cellular function, which is vital for preventing frailty related to cellular decline (48). Regular exercise activates the AMPK signaling pathway in muscle cells, stimulates mitochondrial biogenesis, enhances energy metabolism efficiency, and improves muscle strength and endurance. Muscle contractions triggered by exercise promote the proliferation and differentiation of satellite cells, aiding muscle tissue repair and regeneration, and reducing muscle mass loss associated with frailty (49). Adequate sleep increases growth hormone secretion, supporting muscle growth and repair while regulating neuroendocrine homeostasis. This prevents the accelerated degeneration of bodily functions due to stress hormone imbalances caused by sleep disorders (50). Maintaining a healthy body weight helps prevent chronic inflammation associated with obesity. Imbalances in inflammatory factors disrupt normal cellular signaling, affecting multiple systems, including muscles and bones. Maintaining an appropriate body weight helps preserve internal environmental stability (51). Avoiding smoking and moderating alcohol intake reduces DNA damage from harmful substances and alleviates cardiovascular stress, lowering the risk of frailty by protecting cells and maintaining function. These factors work synergistically to influence frailty in middle-aged and elderly individuals (52).

The study found that the educational level significantly moderates the protective effect of LE8 against frailty (p = 0.012). The negative correlation between the LE8 score and frailty is most pronounced in the group with an education level above high - school, while the protective effect weakens in the group with an education level below high - school. This difference may stem from the following mechanisms. The highly - educated group has greater health literacy, enabling them to effectively understand and implement the LE8 recommendations, such as following a scientific diet and engaging in regular exercise. Meanwhile, they have better economic and medical resources to optimize health indicators, such as BMI and blood - glucose control. In contrast, the low - educated group may have limited access to knowledge, scarce resources, and be affected by psychosocial factors, resulting in insufficient implementation of LE8 - related behaviors.

The components of a healthy lifestyle outlined in LE8, including a balanced diet, regular exercise, and adequate sleep, have significant positive effects on both physical and mental health. When individuals fail to maintain these aspects of health, physical decline and psychological stress may accumulate concurrently. For instance, lack of exercise can slow metabolism and disrupt neurotransmitter balance, impairing emotional regulation and increasing the risk of depression (53). Once depression develops, it can initiate a series of biological chain reactions. Neurobiologically, depression is associated with impaired neural plasticity, reduced hippocampal volume, and dysregulated secretion of neurotransmitters such as serotonin and dopamine (54). This not only impairs cognitive functions but also diminishes self-management and self-motivation, making it harder to adhere to a healthy lifestyle and further deviating from the LE8 guidelines. Physiologically, depression-induced stress activates the hypothalamic-pituitary-adrenal (HPA) axis, increasing the secretion of stress hormones such as cortisol (55). Chronic exposure to elevated cortisol levels suppresses immune function, accelerates muscle catabolism, and reduces bone density. These physiological changes are key characteristics of frailty. Depression creates a link between LE8 and frailty by disrupting neuroendocrine regulation, diminishing psychological resilience, and impairing physiological homeostasis. This mediates the connection between the two (56), underscoring the need for integrated management of psychological health and lifestyle in middle-aged and elderly individuals.

Adopting a healthy lifestyle is essential for reducing frailty. Previous studies have primarily focused on individual components affecting frailty, without considering the holistic aspects. The LE8 score is a comprehensive, user-friendly assessment tool recently introduced by the AHA. It helps evaluate patients’ optimal health status in clinical settings and guide rehabilitation efforts. Our research indicates a significant correlation between LE8 and frailty in middle-aged and elderly individuals. Improving LE8 markers in the elderly can reduce the occurrence of adverse outcomes. The findings provide a scientific foundation for developing targeted health promotion plans and assisting public health institutions in creating guidelines and interventions tailored to the elderly.

This study offers several advantages. The data is derived from the nationally representative National Health and Nutrition Examination Survey (NHANES) of the United States, which is conducted regularly and covers a broad spectrum of non-hospitalized individuals, ensuring generalizability of the results. The study employs a retrospective cohort design, allowing for the observation of temporal relationships. Through multivariable adjustment and various analytical methods, confounding factors are effectively controlled, revealing causality. The comprehensive LE8 score is used to assess cardiovascular health by integrating multiple factors, offering a holistic perspective. It explores the association between LE8 and frailty, investigates the mediating and combined effects of depression, and includes subgroup and sensitivity analyses, offering a thorough understanding of the complex relationship.

This study has several limitations. First, as a cross-sectional study, while confounding factors are controlled as much as possible, it is difficult to definitively determine the causal relationship between LE8 and frailty. The potential influence of reverse causality and other unknown factors cannot be excluded. Second, the LE8 score and Frailty index rely on self-reported data and limited physical examination indicators, which may introduce measurement errors and information biases, affecting result accuracy. Additionally, the study sample is drawn from a specific U.S. population, limiting its representativeness for other regions and racial groups. Caution is necessary when extrapolating the findings. Future research will explore the relationship between the dynamic LE8 score and the risk of frailty or other causes of mortality through long-term follow-up data from a larger, more diverse population.

## Data Availability

Publicly available datasets were analyzed in this study. This data can be found here: https://www.cdc.gov/nchs/nhanes/index.html.
