# Supplementary material for: The mediating and joint effects of depression in the association between cardiovascular health and frailty in middle-aged and elderly people: evidence from NHANES
Source: Front Psychiatry. 2025 Apr 15;16:1578743. doi: 10.3389/fpsyt.2025.1578743 (PMC12037616; doi:10.3389/fpsyt.2025.1578743)
Supplement: Supplementary file 1 [file SupplementaryFile1.docx]

**Supplementary files**

**Legend:**

**Supplementary Table 1** New “Life’s Essential 8” Metrics for Measurement and Quantitative Assessment of CVH

**Supplementary Table 2** Variables in Frailty Index and Their Respective Scores

**Supplementary Table 3** Survey weighted association of Life’s Essential 8 scores with frailty

**Supplementary Table 4** Survey weighted association of the joint effect of Life’s Essential 8 scores and depression with frailty

**Supplementary Table 5** Survey weighted association of Life’s Essential 8 scores with frailty after Multiple Imputation

**Supplementary Table 6** Survey weighted association of Life’s Essential 8 scores with frailty exclude participants with cancer

**Supplementary Table 7** Survey weighted association of Life’s Essential 8 scores with frailty exclude participants with CVD

**Supplementary Table 8** Survey weighted association of Life’s Essential 8 scores with frailty using a cut-off of 0.25

**Supplementary Table 9** Survey weighted association of Life’s Essential 8 scores with frailty after IPTW balanced

**Supplementary Figure 1** Missing data accounted for the proportion of the total population

**Supplementary Table 1** New “Life’s Essential 8” Metrics for Measurement and Quantitative Assessment of CVH

| **CVH metric** | **Method of measurement** | **Quantification of CVH metric** | |
| --- | --- | --- | --- |
| **Diet** |  | Points | Metrics: DASH diet score or Healthy Eating Index-2015 (for populations) |
|  | dietary intake consistent with a Dietary Approaches to Stop Hypertension (DASH)-style eating pattern was assessed using 2 interviewer-administered 24-hour dietary recalls | 100 | 95th percentile (top/ideal diet) |
|  |  | 80 | 75th–94th percentile |
|  |  | 50 | 50th–74th percentile |
|  |  | 25 | 25th–49th percentile |
|  |  | 0 | 1st–24th percentile (bottom/ least ideal quartile) |
| **Physical activity score** |  | Points | Metrics: minutes of moderate to vigorous physical activity per week |
|  | Self-reported minutes of moderate or vigorous physical activity per week | 100 | ≥150 minutes |
|  |  | 90 | 120-149 minutes |
|  |  | 80 | 90-119 minutes |
|  |  | 60 | 60-89 minutes |
|  |  | 40 | 30-59 minutes |
|  |  | 20 | 1-29 minutes |
|  |  | 0 | 0 minutes |
| **Tobacco/nicotine exposure score** |  | Points | Metrics: Combustible tobacco use or inhaled NDS use; or secondhand smoke exposure |
|  | Self-reported use of cigarettes or inhaled nicotine-delivery system (NDS), or secondhand smoke exposure | 100 | Never smoker |
|  |  | 75 | Former smoker, quit ≥ 5 years |
|  |  | 50 | Former smoker, quit 1–<5 years |
|  |  | 25 | Former smoker, quit <1 y, or currently using inhaled NDS |
|  |  | 0 | Current smoker |
|  |  | Subtract 20 points (unless score is 0) for living with active indoor smoker in home | |
| **Sleep health score** |  | Points | Metrics: Average hours of sleep per night |
|  | Self-reported average  hours of sleep per night | 100 | 7-9 hours |
|  |  | 90 | 9-<10 hours |
|  |  | 70 | 6–<7 hours |
|  |  | 40 | 5–<6 or ≥10 hours |
|  |  | 20 | 4–<5 hours |
|  |  | 0 | < 4 hours |
| **Body mass index score** |  | Points | Metrics: BMI |
|  | Body mass index (BMI) was calculated as the weight in kilograms divided by the square of the height in meters from standardized height and weight measurements. | 100 | <25 kg/m^2^ |
|  |  | 70 | 25.0-29.9 kg/m^2^ |
|  |  | 30 | 30.0-34.9 kg/m^2^ |
|  |  | 15 | 35.0-39.9 kg/m^2^ |
|  |  | 0 | ≥ 40.0 kg/m^2^ |
| **Blood lipid score** |  | Points | Metrics: Non-HDL cholesterol |
|  | Non-high-density lipoprotein (non-HDL) cholesterol was calculated by total cholesterol minus HDL cholesterol. | 100 | <130 mg/dL |
|  |  | 60 | 130-159 mg/dL |
|  |  | 40 | 160-189 mg/dL |
|  |  | 20 | 190-219 mg/dL |
|  |  | 0 | ≥ 220 mg/dL |
|  |  | If drug-treated level, subtract 20 points | |
| **Glucose score** |  | Points | Metrics: FBG or HbA1c |
|  | hemoglobin A1c (HbA1c) was measured by high-performance liquid chromatography methods.  Fasting blood glucose (FBG) was measured by standard methods. | 100 | No history of diabetes and  FBG <100 mg/dL (or HbA1c <5.7 %) |
|  |  | 60 | No diabetes and FBG 100–125 100 mg/dL (or HbA1c 5.7–6.4%) |
|  |  | 40 | Diabetes with HbA1c <7.0 % |
|  |  | 30 | Diabetes with HbA1c 7.0–7.9 % |
|  |  | 20 | Diabetes with HbA1c 8.0–8.9 % |
|  |  | 10 | Diabetes with Hb A1c 9.0–9.9 % |
|  |  | 0 | Diabetes with HbA1c ≥10.0 % |
| **Blood pressure score** |  | Points | Metrics: Systolic and diastolic BPs |
|  | The average of all available blood pressure (BP) measurements was used to calculate systolic and diastolic BP. | 100 | <120/<80 mm Hg (optimal) |
|  |  | 75 | 120-129/<80 mm Hg (elevated) |
|  |  | 50 | 130-139 or 80-89 mm Hg (stage 1 hypertension) |
|  |  | 25 | 140-159 or 90-99 mm Hg |
|  |  | 0 | ≥ 160 or ≥ 100 mm Hg |
|  |  | Subtract 20 points (unless score is 0) if treated level | |

**Supplementary Table 2** Variables in Frailty Index and Their Respective Scores

| **Items** | **scores** |
| --- | --- |
| **Cognition** |  |
| 1.experience confusion/memory problems | yes=1, no=0 |
| **Dependence** |  |
| 2.managing money difficulty | no difficulty=0, Some difficulty=0.33, much difficulty=0.66, unable to do=1 |
| 3.walking for a quarter mile difficulty |  |
| 4.walking up ten steps difficulty |  |
| 5.stooping, crouching, kneeling difficulty |  |
| 6.lifting or carrying difficulty |  |
| 7.house chore difficulty |  |
| 8.preparing meals difficulty |  |
| 9.standing up from armless chair difficulty |  |
| 10.getting in and out of bed difficulty |  |
| 11.using fork, knife, drinking from cup difficulty |  |
| 12.dressing yourself difficulty |  |
| 13.standing for long periods difficulty |  |
| 14.grasp/holding small objects difficulty |  |
| 15.attending social event difficulty |  |
| 16.leisure activity at home difficulty |  |
| 17.push or pull large objects difficulty |  |
| **Depressive Conditions** |  |
| 18.have little interest in doing things | nearly every day = 1, more than half the days = 0.66, several days = 0.33, no =0 |
| 19.feeling down, depressed, or hopeless |  |
| 20.trouble sleeping or sleeping too much |  |
| 21.feeling tired or having little energy |  |
| 22.poor appetite or overeating |  |
| 23.feeling bad about yourself |  |
| 24.trouble concentrating on things |  |
| **Comorbidities** |  |
| 25.doctor ever said you had arthritis | yes = 1, no = 0 |
| 26.ever told you had thyroid problem |  |
| 27.ever told you had chronic bronchitis |  |
| 28.ever told you had cancer or malignancy |  |
| 29.ever told had congestive heart failure |  |
| 30.ever told you had coronary heart disease |  |
| 31.ever told you had angina/angina pectoris |  |
| 32.ever told you had heart attack |  |
| 33.ever told you had a stroke |  |
| 34.ever told you had high blood pressure |  |
| 35.doctor told you have diabetes | yes = 1, borderline=0.5, no =0 |
| 36.ever told you had weak/failing kidneys | yes = 1, no =0 |
| 37.urine leakage bother you? | greatly = 1, very much =0.75, somewhat= 0.5, only a little = 0.25, no=0 |
| **Hospital and Care** |  |
| 38.general health condition | excellent, very good, good = 0, fair, poor = 1 |
| 39.health now compared with 1 year ago | worse =1, better = 0 |
| 40.overnight hospital patient in last year | yes =1, no =0 |
| 41.times receive healthcare over past year | no=0, 1-4=0.5, ≥5 =1 |
| 42.number of prescription medicines taken | no =0, 1-4=0.5, ≥5 =1 |
| **Physical Anthropometry** |  |
| 43.body mass index (kg/m^2^) | <18.5, ≥30=1  ≥25, <30=0.5  ≥18.5, ＜25=0 |
| **Laboratory values** |  |
| 44.glycohemoglobin (%) | 0%-5.7%=0, >5.7%=1 |
| 45.red blood cell count (million cells/ul) | M: ≥4.7, <6.1=0, Other=1  F: ≥4.2, ＜5.4=0, Other =1 |
| 46.hemoglobin (g/dl) | M: ≥13.5, <18 =0, Other =1  F: ≥12, <16 =0, Other=1 |
| 47.red cell distribution width (%) | ≥11.6, <14.6=0, Other=1 |
| 48.lymphocyte percent (%) | ≥20, <40=0, Other=1 |
| 49.segmented neutrophils percent (%) | ≥40, <80=0, Other=1 |

**Supplementary Table 3** Survey weighted association of Life’s Essential 8 scores with frailty

| LE8 | Model 1 | | Model 2 | | Model 3 | |
| --- | --- | --- | --- | --- | --- | --- |
|  | OR(95%CI) | P | OR(95%CI) | P | OR(95%CI) | P |
| Low (<50) | 1(Reference) |  | 1(Reference) |  | 1(Reference) |  |
| Moderate (50-80) | 0.28(0.24-0.33) | <0.001 | 0.34(0.29-0.40) | <0.001 | 0.49(0.40-0.58) | <0.001 |
| High (≥80) | 0.06(0.04-0.09) | <0.001 | 0.09(0.06-0.13) | <0.001 | 0.21(0.13-0.33) | <0.001 |
| P for trend |  | <0.001 |  | <0.001 |  | <0.001 |
| LE8 score | 0.53(0.49-0.57) | <0.001 | 0.57(0.54-0.62) | <0.001 | 0.68(0.63-0.74) | <0.001 |

Notes: Model 1 was unadjusted; Model 2 was adjusted for age, gender, ethnicity, education,marital status and PIR ; Model 3 was additionally adjusted for CVD, hypertension, cancer, drinking and diabetes. LE8 score: Life’s Essential 8 scores, as a continuous variable, calculated per 10 points increase.

**Supplementary Table 4** Survey weighted association of the joint effect of Life’s Essential 8 scores and depression with frailty

| LE8 | Model 1 | | Model 2 | | Model 3 | |
| --- | --- | --- | --- | --- | --- | --- |
|  | OR(95%CI) | P | OR(95%CI) | P | OR(95%CI) | P |
| No depression | | | | | | |
| Low (<50) | 1(Reference) |  | 1(Reference) |  | 1(Reference) |  |
| Moderate (50-80) | 0.31(0.27-0.36) | <0.001 | 0.34(0.29-0.40) | <0.001 | 0.49(0.40-0.60) | <0.001 |
| High (≥80) | 0.07(0.04-0.10) | <0.001 | 0.09(0.06-0.14) | <0.001 | 0.21(0.13-0.34) | <0.001 |
| P for trend |  | <0.001 |  | <0.001 |  | <0.001 |
| LE8 score | 0.56(0.52-0.60) | <0.001 | 0.59(0.55-0.63) | <0.001 | 0.71(0.65-0.77) | <0.001 |
| Depression | | | | | | |
| Low (<50) | 1(Reference) |  | 1(Reference) |  | 1(Reference) |  |
| Moderate (50-80) | 0.35(0.17-0.73) | 0.006 | 0.35(0.17-0.74) | 0.006 | 0.59(0.27-1.25) | 0.162 |
| High (≥80) | 0.18(0.05-0.71) | 0.014 | 0.18(0.05-0.70) | 0.014 | 0.55(0.10-2.99) | 0.486 |
| P for trend |  | 0.002 |  | 0.002 |  | 0.258 |
| LE8 score | 0.58(0.44-0.77) | <0.001 | 0.57(0.43-0.75) | <0.001 | 0.68(0.50-0.93) | 0.018 |
| Joint effect of LE8 and depression | | | | | | |
| Low/Depression | 1(Reference) |  | 1(Reference) |  | 1(Reference) |  |
| Moderate/Depression | 0.35(0.17-0.73) | 0.006 | 0.37(0.18-0.76) | 0.008 | 0.57(0.27-1.23) | 0.152 |
| High/Depression | 0.18(0.05-0.71) | 0.014 | 0.19(0.04-0.87) | 0.032 | 0.56(0.09-3.56) | 0.538 |
| Low/No depression | 0.07(0.03-0.13) | <0.001 | 0.06(0.03-0.12) | <0.001 | 0.05(0.03-0.11) | <0.001 |
| Moderate/No depression | 0.02(0.01-0.04) | <0.001 | 0.02(0.01-0.04) | <0.001 | 0.03(0.01-0.05) | <0.001 |
| High/No depression | 0.00(0.00-0.01) | <0.001 | 0.01(0.00-0.01) | <0.001 | 0.01(0.01-0.03) | <0.001 |

Notes: Model 1 was unadjusted; Model 2 was adjusted for age, gender, ethnicity, education,marital status and PIR ; Model 3 was additionally adjusted for CVD, hypertension, cancer, drinking and diabetes. LE8 score: Life’s Essential 8 scores, as a continuous variable, calculated per 10 points increase.

**Supplementary Table 5** Survey weighted association of Life’s Essential 8 scores with frailty after Multiple Imputation

| LE8 | Model 1 | | Model 2 | | Model 3 | |
| --- | --- | --- | --- | --- | --- | --- |
|  | OR(95%CI) | P | OR(95%CI) | P | OR(95%CI) | P |
| Low (<50) | 1(Reference) |  | 1(Reference) |  | 1(Reference) |  |
| Moderate (50-80) | 0.28(0.24-0.32) | <0.001 | 0.33(0.28-0.38) | <0.001 | 0.45(0.38-0.53) | <0.001 |
| High (≥80) | 0.06(0.04-0.09) | <0.001 | 0.09(0.06-0.13) | <0.001 | 0.19(0.13-0.27) | <0.001 |
| P for trend |  | <0.001 |  | <0.001 |  | <0.001 |
| LE8 score | 0.53(0.50-0.56) | <0.001 | 0.57(0.54-0.61) | <0.001 | 0.66(0.62-0.71) | <0.001 |

Notes: Model 1 was unadjusted; Model 2 was adjusted for age, gender, ethnicity, education,marital status and PIR ; Model 3 was additionally adjusted for CVD, hypertension, cancer, drinking and diabetes. LE8 score: Life’s Essential 8 scores, as a continuous variable, calculated per 10 points increase.

**Supplementary Table 6** Survey weighted association of Life’s Essential 8 scores with frailty exclude participants with cancer

| LE8 | Model 1 | | Model 2 | | Model 3 | |
| --- | --- | --- | --- | --- | --- | --- |
|  | OR(95%CI) | P | OR(95%CI) | P | OR(95%CI) | P |
| Low (<50) | 1(Reference) |  | 1(Reference) |  | 1(Reference) |  |
| Moderate (50-80) | 0.26(0.22-0.31) | <0.001 | 0.31(0.26-0.38) | <0.001 | 0.47(0.38-0.58) | <0.001 |
| High (≥80) | 0.05(0.03-0.08) | <0.001 | 0.08(0.05-0.12) | <0.001 | 0.19(0.11-0.30) | <0.001 |
| P for trend |  | <0.001 |  | <0.001 |  | <0.001 |
| LE8 score | 0.52(0.48-0.56) | <0.001 | 0.56(0.52-0.61) | <0.001 | 0.67(0.62-0.74) | <0.001 |

Notes: Model 1 was unadjusted; Model 2 was adjusted for age, gender, ethnicity, education,marital status and PIR ; Model 3 was additionally adjusted for CVD, hypertension, cancer, drinking and diabetes. LE8 score: Life’s Essential 8 scores, as a continuous variable, calculated per 10 points increase.

**Supplementary Table 7** Survey weighted association of Life’s Essential 8 scores with frailty exclude participants with CVD

| LE8 | Model 1 | | Model 2 | | Model 3 | |
| --- | --- | --- | --- | --- | --- | --- |
|  | OR(95%CI) | P | OR(95%CI) | P | OR(95%CI) | P |
| Low (<50) | 1(Reference) |  | 1(Reference) |  | 1(Reference) |  |
| Moderate (50-80) | 0.31(0.25-0.38) | <0.001 | 0.38(0.31-0.46) | <0.001 | 0.50(0.40-0.63) | <0.001 |
| High (≥80) | 0.07(0.04-0.12) | <0.001 | 0.08(0.05-0.12) | <0.001 | 0.21(0.13-0.35) | <0.001 |
| P for trend |  | <0.001 |  | <0.001 |  | <0.001 |
| LE8 score | 0.55(0.51-0.59) | <0.001 | 0.60(0.55-0.64) | <0.001 | 0.68(0.62-0.74) | <0.001 |

Notes: Model 1 was unadjusted; Model 2 was adjusted for age, gender, ethnicity, education,marital status and PIR ; Model 3 was additionally adjusted for CVD, hypertension, cancer, drinking and diabetes. LE8 score: Life’s Essential 8 scores, as a continuous variable, calculated per 10 points increase.

**Supplementary Table 8** Survey weighted association of Life’s Essential 8 scores with frailty using a cut-off of 0.25

| LE8 | Model 1 | | Model 2 | | Model 3 | |
| --- | --- | --- | --- | --- | --- | --- |
|  | OR(95%CI) | P | OR(95%CI) | P | OR(95%CI) | P |
| Low (<50) | 1(Reference) |  | 1(Reference) |  | 1(Reference) |  |
| Moderate (50-80) | 0.27(0.23-0.31) | <0.001 | 0.32(0.28-0.38) | <0.001 | 0.46(0.39-0.56) | <0.001 |
| High (≥80) | 0.06(0.04-0.09) | <0.001 | 0.09(0.06-0.15) | <0.001 | 0.23(0.14-0.38) | <0.001 |
| P for trend |  | <0.001 |  | <0.001 |  | <0.001 |
| LE8 score | 0.53(0.49-0.57) | <0.001 | 0.57(0.54-0.62) | <0.001 | 0.68(0.63-0.74) | <0.001 |

Notes: Model 1 was unadjusted; Model 2 was adjusted for age, gender, ethnicity, education,marital status and PIR ; Model 3 was additionally adjusted for CVD, hypertension, cancer, drinking and diabetes. LE8 score: Life’s Essential 8 scores, as a continuous variable, calculated per 10 points increase.

**Supplementary Table 9** Survey weighted association of Life’s Essential 8 scores with frailty after IPTW balanced

| LE8 | Model 1 | | Model 2 | | Model 3 | |
| --- | --- | --- | --- | --- | --- | --- |
|  | OR(95%CI) | P | OR(95%CI) | P | OR(95%CI) | P |
| Low (<50) | 1(Reference) |  | 1(Reference) |  | 1(Reference) |  |
| Moderate (50-80) | 0.59(0.55-0.63) | <0.001 | 0.57(0.54-0.61) | <0.001 | 0.51(0.47-0.54) | <0.001 |
| High (≥80) | 0.28(0.26-0.30) | <0.001 | 0.27(0.25-0.29) | <0.001 | 0.22(0.21-0.24) | <0.001 |
| P for trend |  | <0.001 |  | <0.001 |  | <0.001 |
| LE8 score | 0.71(0.70-0.72) | <0.001 | 0.71(0.70-0.72) | <0.001 | 0.70(0.69-0.72) | <0.001 |

Notes: Model 1 was unadjusted; Model 2 was adjusted for age, gender, ethnicity, education,marital status and PIR ; Model 3 was additionally adjusted for CVD, hypertension, cancer, drinking and diabetes. LE8 score: Life’s Essential 8 scores, as a continuous variable, calculated per 10 points increase.

**
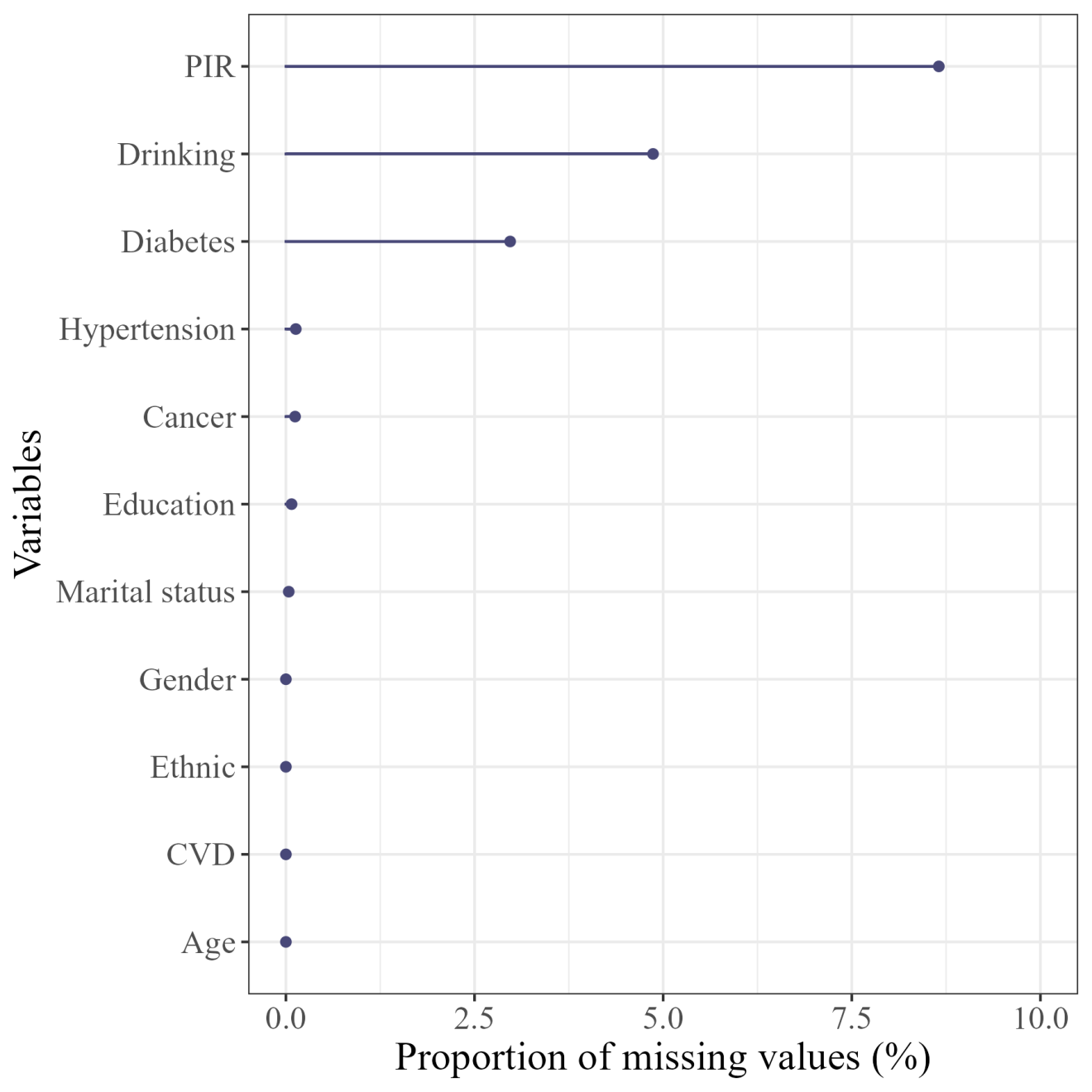
**

**Supplementary Figure 1** Missing data accounted for the proportion of the total population
